# Baseline Correction of the Human ^1^H MRS(I) Spectrum Using T_2_* Selective Differential Operators in the Frequency Domain

**DOI:** 10.3390/metabo12121257

**Published:** 2022-12-14

**Authors:** Sang-Han Choi, Yeun-Chul Ryu, Jun-Young Chung

**Affiliations:** 1 Center for Neuroscience Imaging Research, IBS, N Center, Sungkyunkwan University, Seobu-ro 2066, Jangan-gu, Suwon 16419, Republic of Korea; 2 Department of Radiological Science, College of Health Science, Gachon University, 191 Hambangmoe-ro, Yeonsu-gu, Incheon 21936, Republic of Korea; 3 Department of Neuroscience, College of Medicine, Gachon University, 21, Namdong-daero 774 beon-gil, Namdong-gu, Incheon 21565, Republic of Korea

**Keywords:** MRS(I), T_2_* selective filter, differential filter, baseline correction, water suppression, fat suppression

## Abstract

The baseline distortion caused by water and fat signals is a crucial issue in the ^1^H MRS(I) study of the human brain. This paper suggests an effective and reliable preprocessing technique to calibrate the baseline distortion caused by the water and fat signals exhibited in the MRS spectral signal. For the preprocessing, we designed a T_2_* (or linewidth within the spectral signal) selective filter for the MRS(I) data based on differential filtering within the frequency domain. The number and types for the differential filtering were determined by comparing the T_2_* selectivity profile of each differential operator with the T_2_* profile of the metabolites to be suppressed within the MRS(I) data. In the performance evaluation of the proposed differential filtering, the simulation data for MRS spectral signals were used. Furthermore, the spectral signal of the human ^1^H MRSI data obtained by 2D free induction decay chemical shift imaging with a typical water suppression technique was also used in the performance evaluation. The absolute values of the average of the filtered dataset were quantitatively analyzed using the LCModel software. With the suggested T_2_* selective (not frequency selective) filtering technique, in the simulated MRS data, we removed the metabolites from the simulated MRS(I) spectral signal baseline distorted by the water and fat signal observed in the most frequency band. Moreover, in the obtained MRSI data, the quantitative analysis results for the metabolites of interest showed notable improvement in the uncertainty estimation accuracy, the CRLB (Cramer-Rao Lower Bound) levels.

## 1. Introduction

The baseline of a common ^1^H MRS(I) spectral signal from the human brain is mostly distorted by water and fat signals unless using a technique to suppress them [1]. The MRS(I) signal from water molecules spread all over the brain is significantly larger than the MRS(I) signal from metabolites of interest in the brain. Meanwhile, the MRS(I) signal from fat is smaller than that of water, but a relatively larger signal is observed near the scalp. Even in this case, however, the amount of the signal is considerably larger than that of metabolites of interest. Furthermore, the fat signal, which has a small T_2_*, is observed as a signal with a large linewidth within the spectral signal, and it distorts the baseline shape of the spectral signal in the frequency band of the metabolites of interest.

The baseline distortion caused by the water and fat signals causes an inaccurate quantitative analysis of the metabolites in the MRS(I) data. Therefore, it is crucial to suppress these signals in the ^1^H MRS(I) imaging technique, and various imaging techniques are being used for this purpose. Water suppression via the enhancement of T_1_ effects (WET) and variable power RF pulses with optimized relaxation delays (VAPOR) are some of the representative imaging techniques [2,3]. In both of these techniques, the water signal is pre-saturated into chemical shift selective (CHESS) RF pulses with narrow frequency bandwidths before the localization RF pulse is applied.

Meanwhile, the fat signal is suppressed using the outer volume suppression technique, pre-saturating the signals in the scalp area where the fat signal is usually mostly located [4]. In the case of MRSI using point-resolved spectroscopy (PRESS), which suppresses the fat signal by using selective RF pulses and a crusher gradient, the resonant frequency of the fat signal is set after refocusing the pulse [5]. PRESS-MRSI can also minimize fat artifacts by avoiding excitation of the scalp regions. Furthermore, the water and fat signals are suppressed using frequency selective RF inversion pulses surrounded by spoiler gradient pulses of opposite signs [6].

The water and fat signals in ^1^H MRS(I) can also be suppressed using preprocessing techniques, which include the convolution filtering method on the time-domain of the MRS(I) data [7,8,9]. The band-pass filtering method using a finite impulse response (FIR) filter [10,11] and the singular value decomposition (SVD)-based method [12,13] are examples. However, these previous preprocessing techniques do not consider the simultaneous suppression of both the water and fat signals. Furthermore, their performance has rarely been evaluated with real human brain data using the general tools for quantitative analysis, such as LCModel software [14].

This paper proposes a simple and effective preprocessing technique to calibrate the distortion of the baseline by both water and fat signals in the ^1^H MRS(I) spectral signal simultaneously. The technique is based on the T_2_* selective (not frequency selective) filter through differential filtering of the MRS(I) data in the frequency domain. The accuracy in the proposed method’s quantitative analysis was evaluated by the LCModel software using real human brain MRS(I) data.

## 2. Materials and Methods

### 2.1. Real, Imaginary, and Absolute Component of the MRS Spectral Signal

When the initial phase offset of a signal is 0°, the MRS signal in the time-domain of a single metabolite is expressed in the form of a damped complex exponential such as in Equation (1).

(1) $$S\left(t\right)=S\left(0\right)e^{-2\pi iFt-t/T_{2}^{*}}$$

where “*S*(*t*)” indicates the MRS signal in time-domain, “*S*(0)” is the initial signal intensity in the free induction decay (FID), and “F” is the resonant frequency for the metabolite signal in the rotating frame. Equation (1) is changed into the frequency domain through a Fourier transform. Equations (2)–(4) express the composition of the real, imaginary, and absolute values, each transformed into frequency domains (see Supplementary Equation (S1)); “*f*”, “*BW*”, and “*C*” in Equations (2)–(4) represent the frequency, receive-bandwidth in the data acquisition, and 2πT_2_* of the metabolite.

(2) $$R\left(f\right)=BW\cdot S\left(0\right)\cdot T_{2}^{*}/(1+C^{2}\left(f-F{)}^{2}\right)$$

(3) $$I\left(f\right)=BW\cdot S\left(0\right)\cdot T_{2}^{*}C\left(f-F\right)/(1+C^{2}\left(f-F{)}^{2}\right)=R\left(f\right)C\left(f-F\right)$$

(4) $$A\left(f\right)=R\left(f\right)\sqrt{1+C^{2}{\left(f-F\right)}^{2}}=R\left(F\right)/\sqrt{1+C^{2}{\left(f-F\right)}^{2}}$$

The linewidth within the metabolite’s spectral signal is directly correlated with the T_2_* of the metabolite signal in MRS(I) data. The linewidth within the real and absolute component of the spectral signal derived from Equation (2) and Equation (4) is *1*/(πT_2_*) and $\sqrt{3}$/(πT_2_*), respectively.

### 2.2. Differential Filter in the Frequency Domain and Their T_2_* Selectivity in the MRS Data

The spectral signal of the absolute value filtered by the differential operator can be derived from Equations (2) and (3). The absolute value when f is F at the spectral signal that is filtered by differential operator [−1, 1], [−1, 2, −1], and DFZn [−1, ‘n’, 1] (the number of 0 within the filter is 2 × ‘n’−1) is expressed in Equation (5), (7) (see Supplementary Equations (S2) and (S3)), and Equation (9) (see Supplementary Equations (S4) and (S5)). Additionally, the approximated spectral signal of the absolute value filtered by [−1, 1], [−1, 2, −1], and [−1, ‘n’, 1] is Equations (6), (8), and (10), respectively (see Supplementary Equations (S2)–(S5)).

(5) $$AD1\left(F\right)=R\left(F\right)\cdot C/\sqrt{1+C^{2}}$$

(6) $$\sim AD1\left(f\right)=R\left(f\right)\cdot C/\sqrt{1+C^{2}}$$

(7) $$AD2\left(F\right)=R\left(F\right)\cdot C^{2}/\left(1+C^{2}\right)\;$$

(8) $$AD1\left(F\right)=R\left(F\right)\cdot C/\sqrt{1+C^{2}}$$

(9) $$ADZn\left(F\right)=R\left(F\right)\cdot 2nC/\left(1+n^{2}C^{2}\right)\;$$

(10) $$\sim ADZn\left(f\right)=R\left(f\right)\cdot 2nC/\left(1+n^{2}C^{2}\right)$$

According to Equations (5)–(10), the differential filtering function in the frequency domain is the T_2_* (or linewidth within the spectral signal) dependent function. Furthermore, from Equations (6), (8), and (10), the linewidth of the approximated spectral signal of the differential filtered absolute value (DFAV) is basically the same as that of the spectral signal of the unfiltered real value (UFRV).

**Figure 1:**
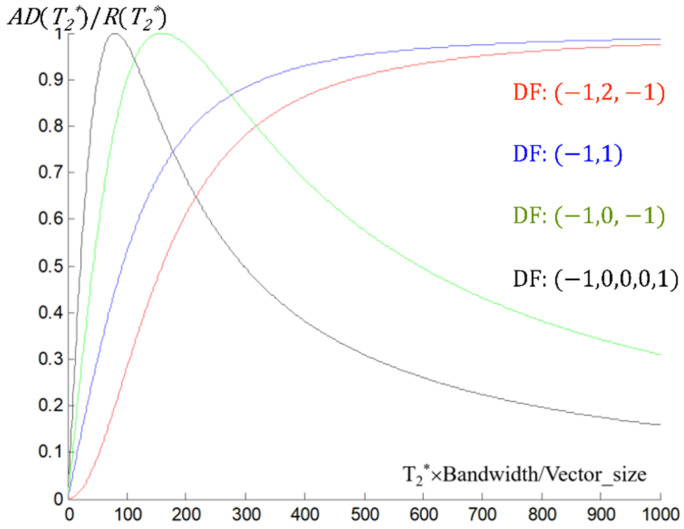
Graphs representing the ratio between the DFAV (filtered by the differential operator, [−1, 2, −1], [−1, 1], [−1, 0, 1], [−1, 0, 0, 0, 1] in the frequency domain) and UFRV according to the T_2_* when the frequency is F at the spectral signal; DF, differential filter; DFAV, differential filtered absolute value; F, resonant frequency for the metabolite signal; UFRV, un-filtered real value.

Figure 1 shows the ratio of the DFAV (*AD*(T_2_*)) and UFRV (*R*(T_2_*)) when the frequency is F at the spectral signal, based on the Equations (5)–(7). The *x*-axis of the graph is T_2_*BW/vector_size (or T_2_*/acquisition_time), and the *y*-axis represents the ratio between DFAV and UFRV when the frequency is F. The graphs in Figure 1 shows a T_2_* selectivity of each differential operator in the spectral signal, and they indicate the differential filter in the frequency domain works as the T_2_* selective filter of the MRS(I) data.

**Figure 2:**
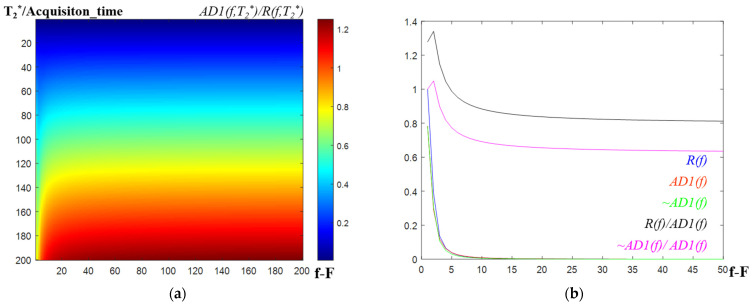
Picture showing that the differential filtering does not distort the shape of the original spectral signal graph (*R*(*f*)) when the differential operator is [−1, 1]. (**a**) The ratio map (according to the T_2_^*^ and f) between the DFAV and UFRV (*AD1*(f, T_2_^*^)/*R*(f, T_2_^*^)). (**b**) Graph of the normalized UFRV (blue), DFAV (red), approximated_DFAV (green). UFRV/DFAV with the black line, and approximated_DFAV/DFAV with the magenta line, when T_2_^*^/acquisition_time is 200. DFAV, differential filtered absolute value; UFRV, un-filtered real value.

Figure 2a shows the ratio map between the DFAV and UFRV when the applied differential operator is [−1, 1], according to the T_2_* and frequency (see Supplementary Equation (S2) for the equation AD1(f, T_2_*)). Figure 2a shows that the mapping result (*AD1*(*f*, T_2_*)/*R*(f, T_2_*)) is smaller than 1 according to the T_2_* when the frequency is F (same data with that of the blue graph in Figure 1), whereas the mapping result becomes larger than 1 when T_2_*/*acquisition_time* is more than 150, depending on the frequency.

Nevertheless, Figure 2b shows that the shape of the differential filtered spectral signal (*AD1*(*f*)) is not mostly distorted from its approximated spectral signal (~*AD1*(*f*)) and un-filtered spectral signal (*R*(*f*)). Figure 2b shows the normalized signal intensity graphs according to the frequency variation when T_2_*/acquisition_time is 200 (lowest line data of the map in Figure 2a) under the condition of Figure 2a. The shape of the graphs indicates that the linewidth of the differential filtered output data (*AD1*(*f*)) is not much different from that of the input real value data (*R*(*f*)). Furthermore, the differential filtered output data (*AD1*(*f*)) are very similar to the approximated *AD1*(*f*) data (red) not evident in the graph.

Figure 1 and Figure 2 indicate that the filtering of the spectral signal by the appropriate differential operator in the frequency domain can selectively suppress a metabolite signal that has a specified T_2_*-band without too much distortion to the other part of the spectral signal.

### 2.3. Preprocessing Procedure

The preprocessing procedure for suppressing the water and fat signals in ^1^H MRS(I) by using differential operators suggested in this paper is as follows: (1) converting the obtained time-domain MRS(I) data to the frequency domain through the Fourier transform; (2) selecting the appropriate band of the T_2_* (or linewidth within the spectral signal) of the metabolites that needs to be suppressed or preserved within the spectral signal; (3) determining the appropriate combination of differential operators (number and types of differential operators combination) automatically by comparing the T_2_* selectivity profile of the differential operators and the selected T_2_*-band; (4) convolving each determined differential operator to the spectral signal; and (5) taking the absolute value of the average of each filtering result from the convolution calculation as the final preprocessed result.

### 2.4. Simulated MRS Data Processing

**Figure 3:**
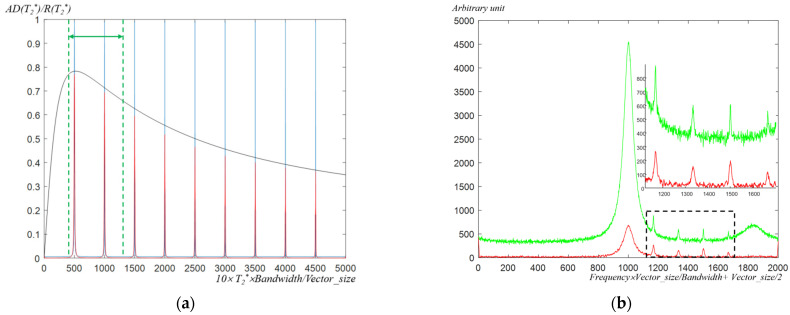
Results of T_2_* selectivity by the differential filtering of the simulation data: (**a**) graphs representing the T_2_* selectivity of the applied differential operators according to the T_2_* when the frequency is F at the spectral signal; spectral signal graphs of the blue line indicate the 9 kinds of the UFRV input signal according to the sequential different F and T_2_*; the black line that shows the ratio between the DFAV and UFRV indicates the expected spectral signal intensity of the output DFAV of each input signal (blue color) when f is F. Spectral signal graphs of the red line show the 9 kinds of the DFAV output signal according to the sequential different F and T_2_*; green dot lines indicate the selected T_2_*-band (40–130 ms); (**b**) spectral signal comparison for input simulation UFRV data (green) and output simulation DFAV data (red); the black dotted box in the graph is the expended view image of the frequency band that shows the metabolites of interest; DFAV, differential filtered absolute value; F, resonant frequency for the metabolite signal; UFRV, un-filtered real value.

In the performance validation of the suggested preprocessing technique, simulated data processing was conducted that is described below. (1) Spectral signals from six types of virtual metabolite were included in the simulation data, and the set-up metabolites and resonant frequencies were water (0 Hz), A (100 Hz), B (200 Hz), C (300 Hz), D (400 Hz), and fat (500 Hz). The T_2_* values and relative signal intensities for the six metabolites were set to (7 ms, 500), (80 ms, 4), (60 ms, 3), (100 ms, 2), (160 ms, 1), and (3 ms, 100), respectively. We added white noise in the simulated data, and their relative signal intensity was set at 0.5. Furthermore, the *BW* and *vector_size* of this simulated data were 1200 and 2000. (2) Based on the T_2_* values of the virtual metabolites, the T_2_*-band that intends to be selectively preserved through differential filtering was selected to the 40–130 ms. (3) The number and type of differential operators according to the determined T_2_*-band were selected automatically by homemade code of Matlab program (MathWorks, Massachusetts, USA). They selected the combination of the differential operators that make the selected T_2_*-band salient (see Figure 3a). The selected differential operators to enhance the selected T_2_*-band (40–130 ms) were {[1, −1], [−1, 0, 1], [−1, 0, 0, 0, 1], [−1, 0, 0, 0, 0, 0, 1], [−1, 0, 0, 0, 0, 0, 0, 0, 1], [−1, 0, 0, 0, 0, 0, 0, 0, 0, 0, 1], and [−1, 0, 0, 0, 0, 0, 0, 0, 0, 0, 0, 0, 1]}. (4) Each determined 7 differential operators were convolved to the simulation spectral signal, separately. (5) We took the absolute value of the average of each filtering result from the convolution calculation as the final preprocessed result.

All of the “Preprocessing Procedure” and “Simulated MRS Data Processing” were performed using homemade code of the MATLAB (Natick, MA, USA), and the code for that is available.

### 2.5. Obtained MRSI Data Processing

Even though differential filtering can improve the spectrum’s appearance to the human eye, the performance in the accuracy of subsequent numerical analysis needs to be validated using real human brain data with the general tools for quantitative analysis [15]. To evaluate the reliability of the suggested technique in human MRSI data, a healthy volunteer participant’s data were obtained using a 7T MRI scanner (Magnetom; Siemens, Erlangen, Germany) and an 8 channel transmit/receive array RF coil. After the participant had been given a complete description of the study, the informed written consent was collected following the Declaration of Helsinki. This study was approved by the Institutional Review Board (IRB).

A 2D FID-chemical shift imaging sequence was used to obtain the MRSI data. This technique is the most frequently used in MR spectroscopic imaging, and it could also best represent the spectroscopic properties. The scan parameters were TR: 1000 ms, FA: 56°, acquisition delay: 1 ms, pulse duration: 1.25 ms, preparation: 2, matrix size: 16 × 16, bandwidth: 2000, vector size: 1024, FOV: 192 mm × 192 mm × 25 mm, delta frequency: −2 ppm, and elliptical phase encoding. During the scan, the axial plane corresponding to the upper part of the corpus callosum was used. We reduced the total time for obtaining the data to 2 m and 31 s using elliptical phase encoding. The WET sequence (bandwidth 80 Hz) was applied to suppress the water signal, but no pre-saturation in the outer volume was performed to suppress the fat signal.

In step (2), the band of the T_2_* (or linewidth within the spectral signal) to be suppressed or preserved within the spectral signal was empirically determined based on the linewidth of the water and fat signals (suppression band), and metabolites signal (preservation band) was seen in the obtained MRSI spectral signal. The remaining preprocessing steps (3)–(5) were the same as those of the “Simulated MRS data Processing”.

The quantitative analysis of the metabolites of interest on the differential filtering result data was conducted by the LCModel software [14]. The complex number data were used in the quantitative analysis of the un-filtered data (that is the standard method in LCModel). In contrast, the absolute value data were applied to the quantitative analysis of the differential filtered data (the result of the preprocessing data is an absolute value).

## 3. Results

### 3.1. Results of the Simulated MRS Data Processing

Figure 3a shows the T_2_* selectivity of the determined combination of differential operators for the simulation data. In the graph, the applied combination of differential operator preserves the data with the T_2_*-band close to 40–130 ms better than the data in other T_2_*-bands. Furthermore, the expected suppression ratio of the spectral signal (black line) from the equations (Equations (5)–(10)) is well-matched with the suppression ratio of the spectral signal in the real differential filtered data (red line graphs).

Within the green graph in Figure 3b, the large signal in the center is the simulated water signal, while the gradual peal signal on the right side is the simulated fat signal. The baseline distortion by the water and fat signal, which has small T_2_* with large signal intensity, in the green graph was removed in the red graph by the low T_2_* signal suppression through the differential filter. Furthermore, the white noise component, which has a seemingly small linewidth within the spectral signal (or large T_2_*) in the green graph, was also reduced in the red graph by the high T_2_* signal suppression through the differential filter. The reduced white noise in the graph indicates the high T_2_*-band suppression filter through the differential filters can be applied to enhance the spectral signal’s signal-to-noise ratio.

From the applied T_2_* selectivity profile of the applied differential operators (Figure 3a), the output simulation MRS data’s signal level can be expected. From Figure 3a, the *AD*(T_2_*)/*R*(T_2_*) is [0.1399, 0.6840, 0.6592, 0.6820, 0.6506, and 0.0606], when the T_2_* is [7, 80, 60, 100, 140, and 3]. Because the peak of the spectral signal is proportional to the signal intensity [500, 4, 3, 2, 1, and 100] and T_2_* of the signal (Equations (1) and (3)), the expected signal level of the output simulation MRS data is proportional to the *signal intensity*·T_2_*·AD(T_2_*)/R(T_2_*). The normalized expected ratio [1.0000, 0.4470, 0.2423, 0.2786, 0.2126, and 0.0371] of the signal peak by this equation is well-matched with the normalized observed ratio [1.0000, 0.4503, 0.2329, 0.2919, 0.2003, and ~0] of the signal peak in the output spectral signal (Figure 3b). The fat signal in the output spectral signal totally disappeared down to the noise level; therefore, we expressed the normalized observed ratio of the fat signal as ~0.

### 3.2. Results of the Obtained MRSI Data Processing

**Figure 4:**
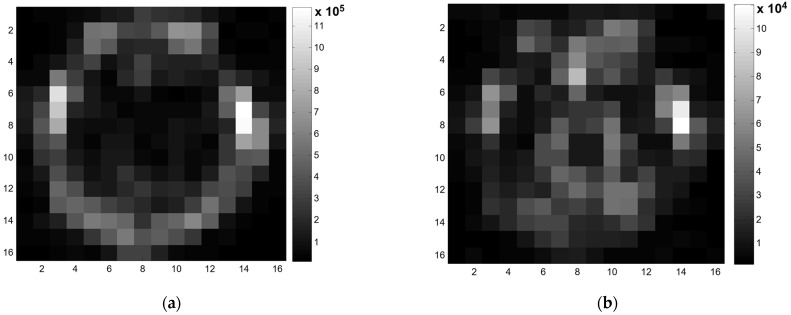
Comparing the MRSI result images before and after differential filtering. For each voxel, the absolute maximum intensity of the spectral signal was mapped: (**a**) the MRSI mapping image before differential filtering; and (**b**) the MRSI mapping image after differential filtering. The scale and high intensity in the brain boundary from fat in Figure 4a were removed in Figure 4b after filtering, revealing signals in the brain area.

**Figure 5:**
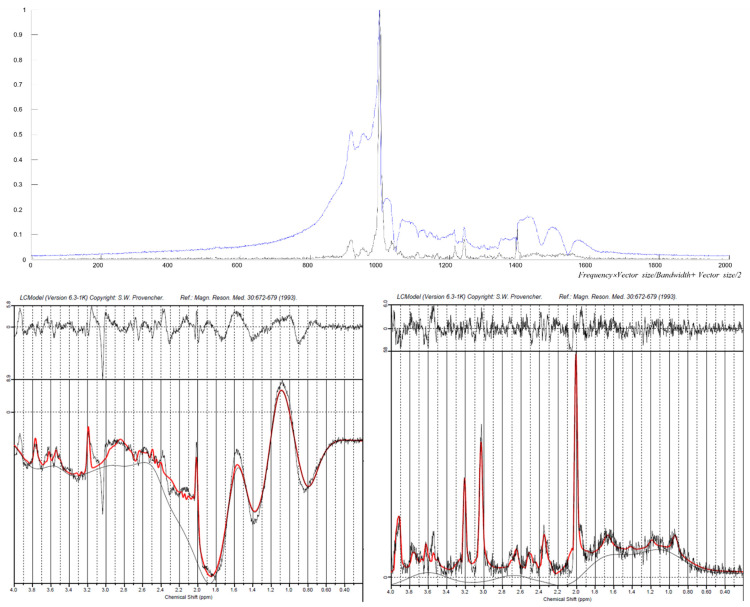
Comparing the spectral signal at the center voxel (8,8) before and after differential filtering. The upper graph indicates the absolute value data before differential filtering (blue) and after differential filtering (black). The *y*-axis is the standardized value of the spectral signal with the maximum, and the *x*-axis is the number of data points. The pictures on the left and right at the bottom are the LCModel analysis data results from un-preprocessed complex number data and differential filtered absolute value data, respectively.

**Figure 6:**
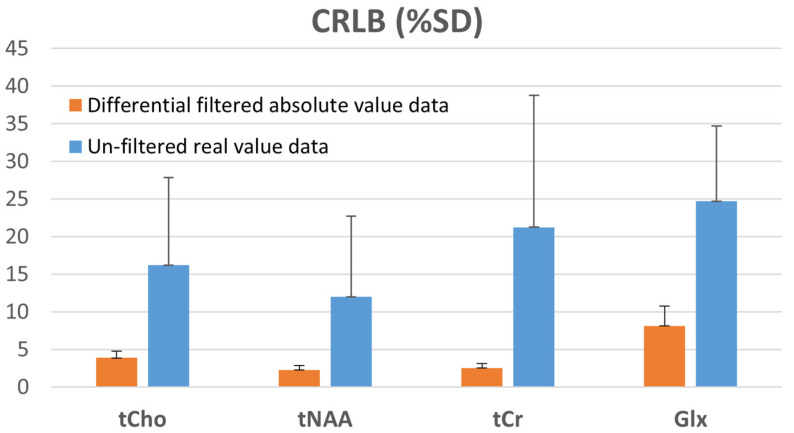
The LCModel quantitative analysis on the original data and the differential filtered data.

Figure 4, Figure 5 and Figure 6 show the filtering result from the obtained ^1^H human MRSI data. We selected T_2_*-band for the differential filtering as 70–200 ms, based on the linewidth of metabolites of interest and water within the obtained MRSI spectral signal. By the selected T_2_*-band, the combination of the differential operators applied in the preprocessing procedure is {[−1, 2, −1], [−1, 1], [−1, 0, 1], and [−1, 0, 0, 0, 1]}.

Figure 4 shows the mapping results of the maximum intensity of the absolute value in the spectral signal per each voxel of the MRSI data. Figure 4a is the result of the original data without differential filtering. By the WET sequence to suppress the water signal without fat suppression in the MRSI acquisition, the bright boundary of the brain in Figure 4a was not from the water signal but from the fat signal. Figure 4b is the result of the preprocessed data with the differential filters. With the water and fat signal suppression through the proposed differential filtering, the bright boundary from the fat signal in Figure 4a was removed, and the bright center of the brain in Figure 4b was not from the fat signal but from the water signal. Furthermore, the original data’s maximum intensity value scale (105) decreased from the differential filtered data (104) to a tenth.

Figure 5 shows the graph comparing the spectral signal in the LCModel software from the central voxel (8,8) before and after differential filtering. From the above graph in Figure 5, the graph for the blue and black color is the absolute value of the spectral signal before and after differential filtering, respectively. The un-preprocessed blue graph shows that the water signal still remained and was large enough to influence the baseline even though the spectral signal was obtained using a sequence with the water suppression technique applied. On the other hand, the differential filtered black graph shows that the baseline distorted by the unsuppressed water signal had been calibrated through the differential filtering procedure.

The lower two pictures in Figure 5 are the graphs of the LCModel analysis results of the corresponding data. The graph on the left, the analyzing result of the original complex number data, shows an unstable baseline (red curve) due to the fat signal being included in the spectral signal. The graph on the right, which is the analyzing result of the differential filtered absolute value data, shows a stable baseline. The black graph in the figure is the input data after being preprocessed by the LCModel, and the red graph is the fitted data from the LCModel. The error graph between input data and fitted data is indicated at the top, and that of the differential filtered data seems more stable than that of the original data.

Figure 6 is the resulting graph of the LCModel quantitative analysis on the original data and the differential filtered data. The metabolites of interest that were quantitatively analyzed were tCho, tNAA, tCr, and Glx; and the quantitative analysis errors (%SD) in 16 voxels (7–10,7–10) located at the center among all voxels were averaged. In all the analyzed metabolites, the differential filtered data shows much smaller analysis errors than the original data in the results.

## 4. Discussion

This paper proposed a differential filtering method in the frequency domain for the MRS(I) signal to calibrate the baseline distortion of water and fat signals in the spectral signal when the water and fat signals have not been fully suppressed. The water and fat signals in ^1^H MRS(I) could be suppressed using preprocessing techniques, such as the convolution filtering method on the time-domain of the MRS(I) data [7,8,9], the band-pass filtering method using a finite impulse response (FIR) filter [10,11], and the singular value decomposition (SVD)-based method [12,13]. Nevertheless, these preprocessing techniques did not consider the simultaneous suppression of the water and fat signals. However, there is no filtering technique that considers both water and fat. Through the LCModel analysis of actual human brain test data and the simulation data testing, this paper verified that the proposed method could be used to calibrate the baseline distortion in the MR spectral signal and demonstrated that the proposed method is stable and effective in enhancing the quantitative accuracy of metabolites’ analysis.

The proposed approach has several potential limitations. First, the determination method of the differential filter in this approach is automatic but not systematic. We determined the combination using a rough arithmetic approach. Therefore, the differential filtering result in this report is not optimal and has a chance to improve performance. Second, the proposed T_2_* selective filtering approach depends on the T_2_* values of the different resonances in the spectrum. This T_2_* dependence makes the method unsuitable for quantitative spectral signal analysis with a fixed T_2_*-band for metabolites of interest regardless of the B_0_ inhomogeneity in the MRS(I) data. Including the determination of the T_2_* (or linewidth within the spectral signal) of the metabolites signal into the processing procedure (see Step (2) in “Preprocessing Procedure”) would solve these issues. Furthermore, the LCModel manual recommends unfiltered complex number data as LCModel input data for accurate uncertainty estimations (CRLBs). Although the performance is better than the standard unfiltered complex number input, our results (Figure 6) based on the filtered absolute value data are not the best for the LCModel.

This method considers the preprocessing procedure. Hence, this technique can be applied with the water and/or fat suppression technique for imaging acquisition. This method applies to the MR spectroscopic data based on a water suppression MR technique, such as WET or VAPOR, and/or fat suppression MR technique, such as outer volume suppression. Then, our method may include the additional suppression of the water and fat components in the spectral signal.

The proposed method preprocesses the frequency domain data; therefore, the differential filtered results data must be applied by a quantitative analysis method based on the frequency domain data (e.g., a quantitative analysis method in LCModel) to achieve an appropriate result. If the results from the proposed preprocessing method are applied using anything other than a quantitative approach based on a filter for time-domain data (e.g., AMARES in JMRUI), the accuracy of the analysis will decrease [16]. Moreover, the proposed method is inappropriate for spectral signals where the water signal is not suppressed. The proposed method can improve the quantitative analysis result of MRSI data obtained without water signal suppression. Nevertheless, the result was not sufficiently stable compared to the MRSI data obtained using the water suppression procedure.

In this study, we described typical, simple approaches using isotropic phantoms containing water, fat, and paramagnetic ions. We can also consider advanced phantoms with anisotropic (laminar and/or capillary) structures filled with various reference mixtures (issue—innovative anisotropic phantoms). Using alternative techniques, such as the Carr–Purcell–Meiboom–Gill sequence, to determine T_2_ distributions, then after subtracting the peaks from water and fat [17,18,19,20,21,22], allows us to return to the raw data and perform the fast Fourier transform (FFT). Additionally, obtaining T_1_ will allow the generation of T_1_/T_2_ maps, providing additional opportunities to separate signals from water and fat [23,24]. They can be related to the issues of geophysical space: “Identification of Proton Populations” and “Overcoming the Barriers of Nanoporous Porosity”. Separating the signal by the diffusion coefficient, diffusion-weighted imaging (DWI), and diffusion tensor imaging (DTI) techniques are also worthy of consideration [25,26,27,28].

The proposed method has several differences with strengths over the conventional preprocessing techniques to suppress the water and fat signals. Firstly, the typical MRS preprocessing method filters the data based on frequency selectivity [7,8,9,10,11,12,13], whereas the suggested method filters the data based on the T_2_* selectivity. Since the proposed method is available for any metabolites regardless of their frequency band, it can suppress the water signal even if the water signal’s resonance frequency in the spectral signal is not precisely 0 Hz. For the same reason, the suggested method can also suppress the fat signal when the resonance frequency is not 0 Hz without any further knowledge relating to the frequency characteristics.

Secondly, the values from the results of the existing spectral signal preprocessing method are mainly complex numbers of data, whereas the result values of the suggested spectral signal preprocessing method are absolute value data. Hence, unlike the other methods, the suggested method does not have shape distortion in the spectral signal due to phase noise. As a result, the suggested method does not need calibration on the phase distortion in the original signal or any distortion that may occur during the preprocessing procedure.

## 5. Conclusions

In conclusion, we proposed the T_2_* selective filter based on the differential filter in the frequency domain in this paper. In addition, through the quantitative analysis of obtained MRSI data, we demonstrated that by exploiting the proposed T_2_* selective filter, the baseline distortion from the water and fat signals in the human ^1^H MRS(I) spectral signal can be corrected.

## Data Availability

Not applicable.

## Supplementary Materials

The following supporting information can be downloaded at: https://www.mdpi.com/article/10.3390/metabo12121257/s1, Supplementary Equation (S1): Profile of the MRS spectrum data; Supplementary Equation (S2): Profile of the differential filter [1, −1] in the Spectrum; Supplementary Equation (S3): Profile of the differential filter [−1, 2, −1] in the Spectrum; Supplementary Equation (S4): Profile of the differential filter [1, 0, −1] in the Spectrum; Supplementary Equation (S5): Profile of the differential filter [1, 0, 0, 0, −1] in the Spectrum.
