# Supplementary material for: Baseline Correction of the Human 1H MRS(I) Spectrum Using T2* Selective Differential Operators in the Frequency Domain
Source: Metabolites. 2022 Dec 14;12(12):1257. doi: 10.3390/metabo12121257 (PMC9787948; doi:10.3390/metabo12121257)
Supplement: Supplementary file 1 [file metabolites-12-01257-s001.zip › metabolites-2049806-supplementary.pdf]

*Supplementary Material*

# Title: Baseline correction of 1H MRS Spectrum using T2* selective differential operators in the frequency-domain

**Sang-Han Choi [1], Yeunchul Ryu [2,*] and Jun-Young Chung [3,*]**

[1]IBS Center for Neuroscience Imaging Research, N Center, Sungkyunkwan University, Seobu-ro 2066, Jangan-gu, Suwon, Republic of Korea. 16419

[2]Department of Neuroscience, College of Medicine, Gachon University, 1198 Kuwol-dong, Namdong-gu, Incheon, Republic of Korea. 405-760

[3]Department of Neuroscience, College of Medicine, Gachon University, 21, Namdong-daero 774beon-gil, Namdong-gu, Incheon, Republic of Korea, 21565

*** Correspondence:** Jun-Young Chung, jychung@gachon.ac.kr

# 1 Supplementary Equations

## 1.1 Supplementary Equation 1. Profile of the MRS spectrum data

**Let,** ‘S(t)’ = complex of the MRS time (t) domain data (Signal)

‘R(f)’ = real of the frequency (f) domain data (Spectrum) of the S(t)

‘I(f)’ = imaginary of the Spectrum of the S(t)

‘A(f)’ = absolute value of the Spectrum of the S(t)

‘F’ = resonant frequency of the considered metabolite

‘BW’ = received bandwidth

‘C’ = $2\times\pi\times T_2^*$

‘LW[X(f)]’ = line width of the Spectrum X

**Put**, initial phase offset of a S(t) is 0

**Then,**

**Equation S1.** $S(t) = S(0)\times e^{-i\times 2\times\pi\times F\times t - t/T_2^*}$

**Equation S2.** $R(f) = BW\times S(0)\times T_2^* / (1 + C^2\times(f-F)^2)$

**Equation S3.** $R(f)/R(F) = 1/(1 + C^2\times(f-F)^2)$

**Equation S4.** $I(f) = BW\times S(0)\times T_2^*\times C\times(f-F)/(1 + C^2\times(f-F)^2) = R(f)\times C\times(f-F)$

**Equation S5.** $A(f) = \sqrt{R(f)^2 + I(f)^2} = R(f)\times\sqrt{1 + C^2\times(f-F)^2} = R(F)/\sqrt{1 + C^2\times(f-F)^2}$

**Equation S6.** $LW[R(f)] = 1/(\pi\times T_2^*)$=2/C

**Equation S7.** $LW[A(f)] = \sqrt{3}/(\pi\times T_2^*)$

⇨ $LW[A(f)] = \sqrt{3}\times LW[R(f)]$

## 1.2 Supplementary Equation 2. Profile of the differential filter [1, -1] in the Spectrum

**Put,** 'Y(f)'=$(1+C^2\times(f+1)^2)\times(1+C^2\times f^2)$

**Then,** $Y(0)=1+C^2$

**Equation S1-1.** $R(f)=BW\times S(0)\times T_2^*/(1+C^2\times(f-F)^2)$

$$=R(F)/(1+C^2\times(f-F)^2)$$

**Equation S1-2.** $R(f+1)=BW\times S(0)\times T_2^*/(1+C^2\times(f-F+1)^2)$

**Equation S2-1.** $RD1(f)=R(f+1)-R(f)\;=-R(F)\times C^2\times(2\times(f-F)+1)/Y(f-F)$

**Equation S3-1.** $ID1(f)=I(f+1)-I(f)=\;R(F)\times C\times[1-(f-F)\times C^2-(f-F)^2\times C^2]/Y(f-F)$

**Equation S4-1.** $AD1(f)=\sqrt{RD1(f)^2+ID1(f)^2}$

$$=\;R(F)\times C\times\sqrt{(C\times[2\times(f-F)+1])^2+(1-(f-F)^2\times C^2-(f-F)\times C^2)^2}\;/Y(f-F)$$

**Equation S4-2.** $AD1(F)=R(F)\times C/\sqrt{1+C^2}$

**Equation S4-3.** $AD1(f)\sim=R(F)\times C/([1+C^2\times(f-F)^2)\times\sqrt{1+C^2}$ , when small C or small f-F

$\sim=R(f)\times C/\sqrt{1+C^2}=R(f)\times AD1(F)/R(F)$, when small C or small f-F

⇨ LW[AD1(f)]~=LW[R(f)] , when small C or small f-F

### 1.3 Supplementary Equation 3. Profile of the differential filter [-1, 2, -1] in the Spectrum

**Put,** ‘Z(f)’=$(1+C^2\times(f-1)^2)\times(1+C^2\times f^2)\times(1+C^2\times(f+1)^2)$

**Then,** $Z(0)=(1+C^2)^2$

**Equation S1-1.** $R(f-1)=BW\times S(0)\times T_2^* / (1+C^2\times(f-F-1)^2)$

**Equation S2-1.** $RD2(f)=-R(f+1)+2\times R(f)-R(f-1)$

$$= R(F)\times 2\times C^2\times(1+C^2\times[1-3\times(f-F)^2])/Z(f-F)$$

**Equation S2-2.** $RD2(F)=R(F)\times 2\times C^2/(1+C^2)$

**Equation S3-1.** $ID2(f)=-I(f+1)+2\times I(f)-I(f-1)$

$$= R(F)\times 2\times C^3\times(f-F)\times(C^2\times[1-5\times(f-F)^2]-1)/Z(f-F)$$

**Equation S3-2.** $ID2(F)=0$

**Equation S4-1.** $AD2(f)=\sqrt{RD2(f)^2+ID2(f)^2}$

$$=R(F)\times C^2/Z(f-F)\times\sqrt{(1+C^2\times[1-3\times(f-F)^2])^2+[C\times(f-F)\times(C^2\times[1-5\times(f-F)^2]-1)]^2}$$

**Equation S4-2.** $AD2(F)=RD2(F)=R(F)\times C^2/(1+C^2)$

**Equation S4-3.** $AD2(f)\sim=R(f)\times(1+C^2\times(f-F)^2)\times C^2/[(1+C^2\times(f-F)^2)\times(1+C^2)^2]\times(1+C^2)$,when small C or small f-F

$\sim=R(f)\times C^2/(1+C^2)=R(f)\times AD2(F)/R(F)$,when small C or small f-F

⇨ LW[AD2(f)]~=LW[R(f)] **,**when small C or small f-F

## 1.4 Supplementary Equation 4. Profile of the differential filter [1, 0,-1] in the Spectrum

**Put,** ‘X1(f)’=$(1+ C^2 \times (f+1)^2) \times (1+ C^2 \times (f-1)^2)$

**Then,** $X1(0)=(1+C^2)^2$

**Equation S1-1.** $RDZ1(f) = R(f+1) - R(f-1)$

$$= -2 \times R(F) \times 2 \times C^2 \times (f-F) / X1(f-F)$$

**Equation S1-2.** $RDZ1(F) = 0$

**Equation S2-1.** $IDZ1(f) = I(f+1) - I(f-1)$

$$= 2 \times R(F) \times C \times (-C^2 \times (f-F)^2 + C^2 + 1) / X1(f-F)$$

**Equation S2-2.** $IDZ1(F) = 2 \times R(F) \times C / (1 + C^2)$

**Equation S3-1.** $ADZ1(f) = \sqrt{RDZ1(f)^2 + IDZ1(f)^2}$

$$= 2 \times R(F) \times C \times \sqrt{(2 \times C \times (f-F))^2 + (-(C \times (f-F))^2 + 1 \times C^2 + 1)^2} / X1(f-F)$$

**Equation S3-2.** $ADZ1(F) = 2 \times R(F) \times C / (1 + 1 \times C^2)$

**Equation S3-3.** $ADZ1(f) \sim= 2 \times R(F) \times C \times /[(1 + C^2 \times (f-F)^2) \times 1 \times (1 + C^2)]$ , when small C or small f-F

$\sim= 2 \times R(F) \times C / (1 + 1 \times C^2) = R(f) \times ADZ1(F) / R(F)$, when small C or small f-F

⇨ LW[AZD1(f)]~=LW[R(f)] , when small C or small f-F

## 1.5 Supplementary Equation 5. Profile of the differential filter [1, 0, 0, 0,-1] in the Spectrum

**Put,** ‘X2(f)’=$(1+ C^2\times(f+2)^2)\times( 1+ C^2\times(f-2)^2)$

**Then,** $X2(0)=(1+4\times C^2)^2$

**Equation S1-1.** $RDZ2(f)=R(f+2)-R(f-2)$

$$=-4\times R(F)\times 2\times C^2\times(f-F)/X2(f-F)$$

**Equation S1-2.** $RDZ2(F)=0$

**Equation S2-1.** $IDZ2(f)=I(f+2)-I(f-2)$

$$=4\times R(F)\times C\times(-C^2\times(f-F)^2+4\times C^2+1)/X2(f-F)$$

**Equation S2-2.** $IDZ2(F)=4\times R(F)\times C/(1+4\times C^2)$

**Equation S3-1.** $ADZ2(f)=\sqrt{RDZ2(f)^2+IDZ2(f)^2}$

$$= 4\times R(F)\times C\times\sqrt{(2\times C\times(f-F))^2+(-(C\times(f-F))^2+4\times C^2+1)^2}/X2(f-F)$$

**Equation S3-2.** $ADZ2(F)=4\times R(F)\times C/(1+4\times C^2)$

**Equation S3-3.** $ADZ2(f)\sim=4\times CR(F)\times\times/[(1+C^2\times(f-F)^2)\times(1+4\times C^2)]$ , when small C or small f-F

$\sim=4\times R(F)\times C/(1+4\times C^2)=R(f)\times ADZ2(F)/R(F)$, when small C or small f-F

⇨ LW[AZD2(f)]~=LW[R(f)] , when small C or small f-F
